# MIDLIFE CHRONIC STRESS, PHYSIOLOGICAL DYSREGULATION, AND COGNITION: A LONGITUDINAL MEDIATION ANALYSIS

**DOI:** 10.1093/geroni/igae098.1308

**Published:** 2024-12-31

**Authors:** Uchechi Mitchell

**Affiliations:** University of Illinois Chicago, Chicago, Illinois, United States

## Abstract

The current study investigates how exposure to chronic stressors during mid-life indirectly affects cognitive function through changes in physiological dysregulation. Data come from 4,684 adults ages 51-64 at baseline who participated in the Health and Retirement Study between 2006 and 2018. Mid-life stressors include experiences of everyday discrimination and financial strain, both of which were assessed at baseline only. Physiological dysregulation was assessed as a count of high-risk inflammatory, cardiovascular, and metabolic biomarkers and was assessed every four years. Cognitive function assessed every two years using a modified version of the TICS. A longitudinal mediation analysis was conducted using R and MPLUS. Unadjusted analyses showed that cognitive function decreased over time, while physiological dysregulation significantly increased. The indirect effects of financial strain on cognitive decline were mediated by baseline dysregulation levels and increases in physiological dysregulation over time. On the other hand, the effect of everyday discrimination on cognitive decline was only mediated by its effect on baseline physiological dysregulation. These findings suggest that 1) physiologic vulnerability to financial strain and everyday discrimination may be more salient earlier in the life course setting the stage for changes in cognition later in life, and 2) financial strain in mid-life continues to adversely affect physiological functioning and cognition over time. That said, adjusting for age, gender, race/ethnicity and education fully accounted for the significant direct and indirect effects of financial strain and discrimination on changes in cognition, suggesting a more complex and nuanced relationship between chronic stress, physiological function and cognition.

